# Encoding the Laplace transform of stimulus history using mechanisms for persistent firing

**DOI:** 10.1186/1471-2202-14-S1-P356

**Published:** 2013-07-08

**Authors:** Zoran Tiganj, Karthik H Shankar, Marc W Howard

**Affiliations:** 1 Center for Memory and Brain, Boston University, Boston, MA 02215, USA

## 

Persistent firing has been observed in slice preparations from a variety of brain regions believed to be important in memory [1-3]. Information about a transient stimulus can be reflected in persistently elevated firing rate over many minutes. What computation could persistent firing cells support? In particular the lack of forgetting observed in slice preparations seems difficult to reconcile with the finding that memory degrades over time.

A scale-invariant model for representing past stimuli has been proposed in [4]. This model utilizes two layers of nodes. The first layer is composed of leaky integrators and computes the Laplace transform of the input function. The first layer projects to the second through a linear operator which approximates the inverse Laplace transform so the activity of the second layer at any point of time gives a fuzzy representation of the input history leading up to the present moment. Comparison with behavioral results suggests that the history should be retained over at least a few thousand seconds.

If we assume that each node of the above model corresponds to an individual neuron, the neurons encoding the Laplace transform should respond to a particular stimulus with a firing rate that decays exponentially over time. Critically, in order to encode the Laplace transform we require different rates of decay for different neurons in this layer. Moreover, the longest time constant across neurons determines the longest time scale that can be maintained in representation of the history.

Persistent firing cells [5] are basically perfect integrators. We have been exploring the conditions under which persistently firing cells observed in the slice could be adapted to give rise to a set of leaky integrators whose rate constants are under external control. The basic idea is to modulate the channels responsible for persistent firing using divisive dendritic inhibition [6]. Under these circumstances, constructing a set of cells with different rate constants amounts to providing a set of identical cells with different levels of inhibitory input. We explore the potential of this mechanism using biophysical simulations centered on a calcium-dependent cationic current - Ican and hyperpolarization-activated, voltage and calcium-dependent cationic current - Ih. We also describe a simple integrate and fire model that yields an explicit analytical solution describing the conditions under which persistent firing can be modulated to yield exponential decay.
